# Supplementary figures and images for: mTOR Signaling Pathway Regulates Sperm Quality in Older Men
Source: Cells. 2019 Jun 21;8(6):629. doi: 10.3390/cells8060629 (PMC6627782; doi:10.3390/cells8060629)

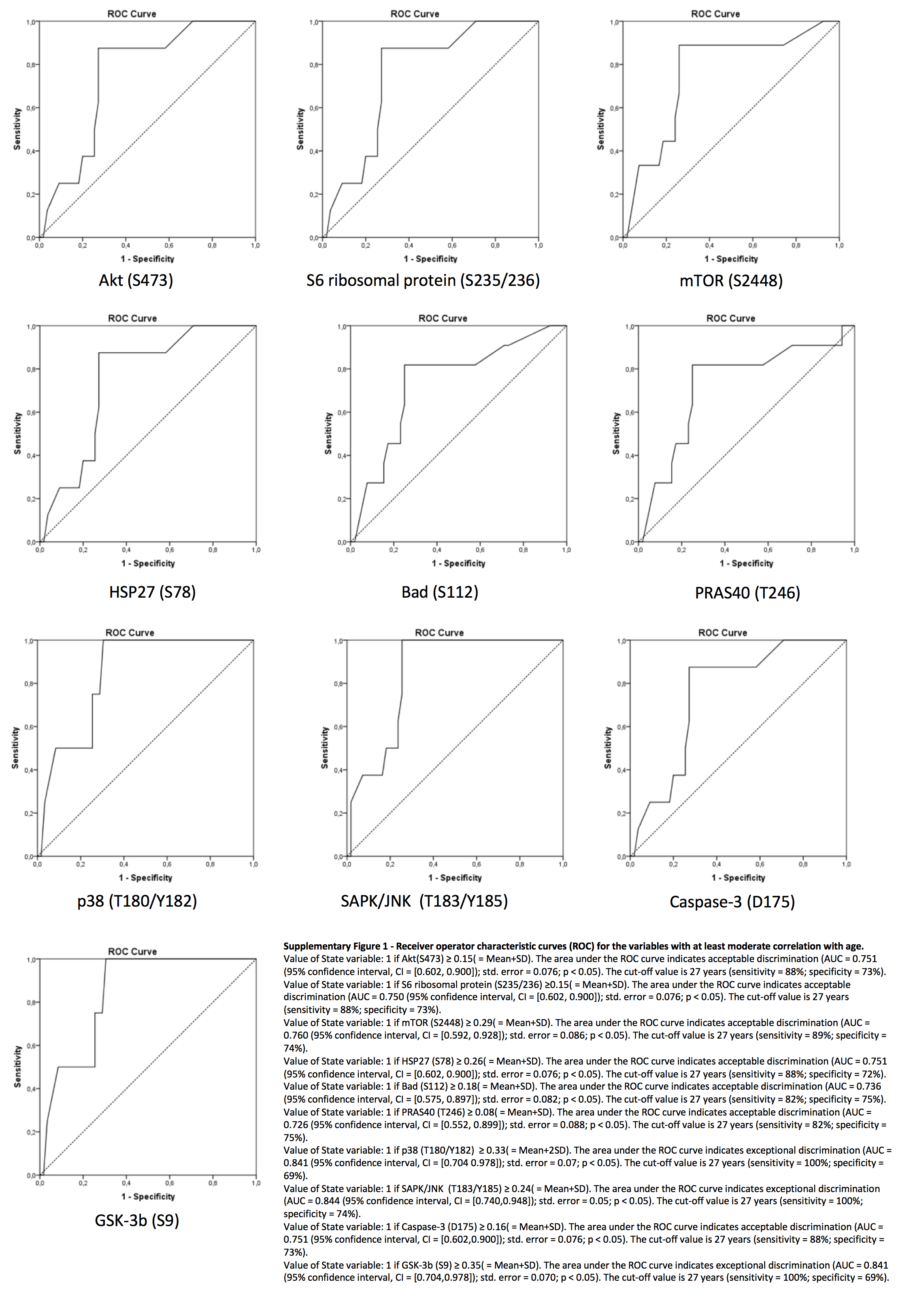

Supplement: Supplementary file 1 [file cells-08-00629-s001.zip › Supplementary Figure 1.tiff]

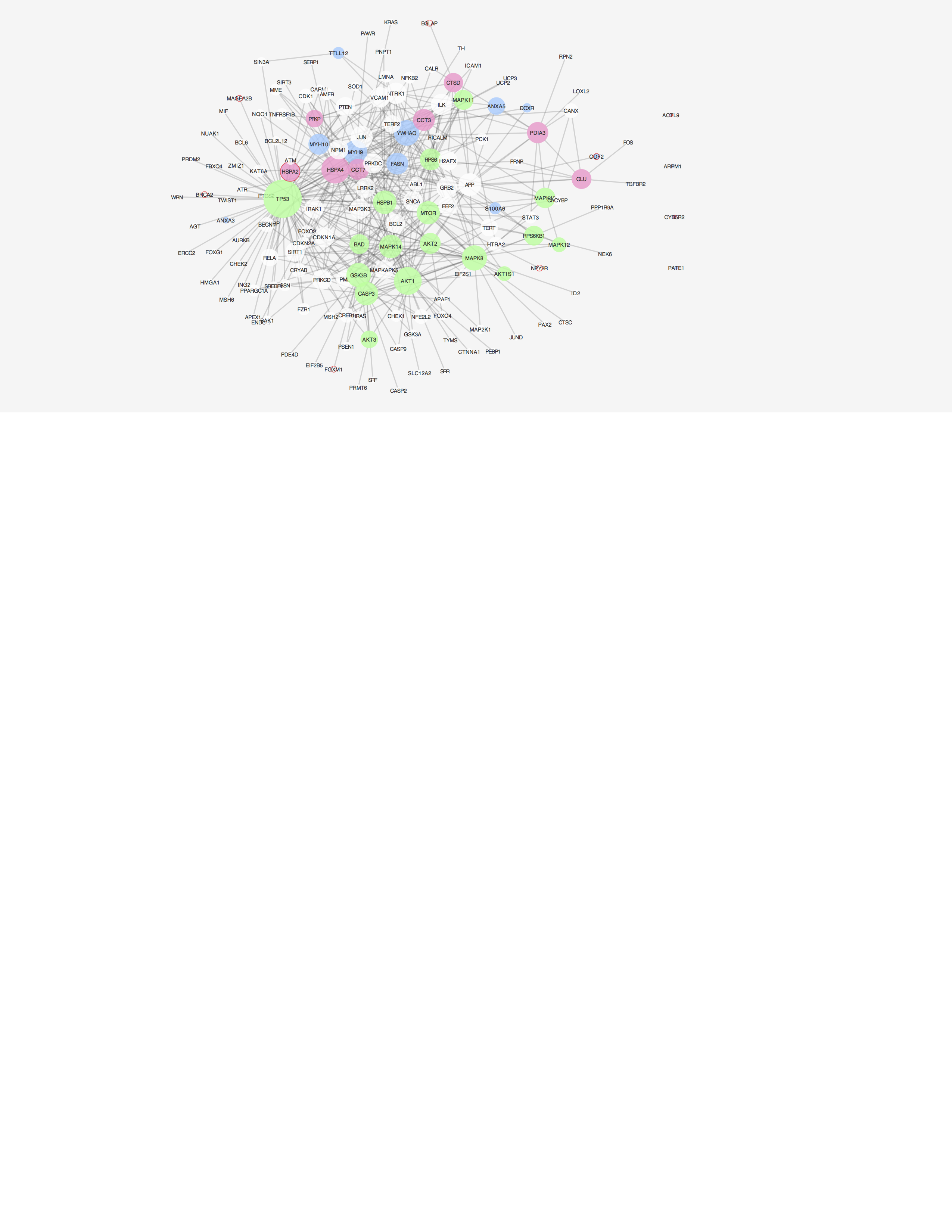

Supplement: Supplementary file 1 [file cells-08-00629-s001.zip › Supplementary Figure 2.tiff]
